# Nanoformulated *Walterinnesia aegyptia* Venom Enhances Therapeutic Outcomes in Experimental Cutaneous Leishmaniasis: A Comparative Study of Hyaluronic Acid and Silver Nanoparticles

**DOI:** 10.3390/nano16100614

**Published:** 2026-05-17

**Authors:** Almaha Al-Aqil, Ibrahim S. Al Nasr, Hana Hakami, Faten Abou El Fadl, Chuanyi Wang, Mona Al-Shammari, Samiah Alotaibi, Sohaialah Alotaibi, Afnan Al-Qurashi, Huda Al-Rashid, Ebtesam Al-Olayan

**Affiliations:** 1 Zoology Department, College of Science, King Saud University, Riyadh 11451, Saudi Arabia; almaha.alaqeel@hotmail.com (A.A.-A.); 443204468@student.ksu.edu.sa (M.A.-S.); samiahalotaibi96@gmail.com (S.A.); 443203299@student.ksu.edu.sa (A.A.-Q.); 2 Department of Biology, College of Science, Qassim University, Buraydah 52571, Saudi Arabia; insar@qu.edu.sa; 3 Polymer Chemistry Department, National Center for Radiation Research and Technology (NCRRT), Egyptian Atomic Energy Authority, Nasr City, P.O. Box 7551, Cairo 11762, Egypt; fatenelramly555@yahoo.com; 4 School of Environmental Science and Engineering, Shaanxi University of Science and Technology, Xi’an 710021, China; cywang@ms.xjb.as.cn; 5 Chair for Infectious Disease Vaccine Research, Zoology Department, College of Science, King Saud University, Riyadh 11451, Saudi Arabia; halmadfaa@ksu.edu.sa

**Keywords:** nanomedicine, drug delivery systems, targeted therapy, biocompatibility, cutaneous leishmaniasis, *Leishmania major*, hyaluronic acid nanoparticles, silver nanoparticles, venom-based therapy, *Walterinnesia aegyptia* venom

## Abstract

Cutaneous leishmaniasis remains a major therapeutic challenge due to drug toxicity, resistance, and limited efficacy against intracellular parasites. This study evaluated the therapeutic potential of nanoformulated *Walterinnesia aegyptia* (WA) venom using hyaluronic acid-based (WA-HA) and silver-based (WA-Ag) nanoparticles. Nanoparticles were synthesized and characterized by scanning and transmission electron microscopy and energy-dispersive X-ray spectroscopy. The antipromastigote activity of crude WA venom was assessed by MTT assay, and apoptosis induction was analyzed using Annexin V-FITC/propidium iodide flow cytometry. In vivo efficacy was evaluated in BALB/c mice infected with *Leishmania major*, with outcomes assessed by lesion progression, biochemical markers, histopathology, and PCR-based parasite detection. WA venom exhibited potent dose-dependent cytotoxicity (IC50 = 26.73 µg/mL) and induced predominantly apoptotic cell death. In vivo, nanoformulated WA significantly enhanced therapeutic outcomes compared with crude venom, with WA-HA achieving near-complete lesion resolution comparable to Amphotericin B. Treatment also reduced parasite burden, normalized liver enzyme levels, and restored hepatic and splenic architecture. These findings demonstrate that nanocarrier-based delivery markedly improves the therapeutic performance and systemic safety of WA venom, highlighting its potential as a promising nanotherapeutic strategy for cutaneous leishmaniasis.

## 1. Introduction

Parasitic diseases continue to represent a major global health burden, particularly in low- and middle-income countries. Among protozoan infections, leishmaniasis is a neglected tropical disease caused by parasites of the genus *Leishmania* [1]. Transmission occurs through the bite of infected female sand flies, resulting in three principal clinical forms: cutaneous (CL), mucocutaneous (MCL), and visceral leishmaniasis (VL) [2].

Cutaneous leishmaniasis (CL) is the most prevalent form and is characterized by localized skin lesions that frequently ulcerate, lead to permanent scarring, and impose substantial social stigma. According to the World Health Organization (WHO), hundreds of thousands of new cases are reported annually, with CL accounting for the majority of infections worldwide [3]. Although CL has lower mortality than VL, it constitutes a significant public health burden due to its chronic course, disfiguring lesions, and limited access to effective treatment in endemic regions.

*Leishmania* parasites are obligate intracellular pathogens that differentiate from promastigotes in the sand fly vector into amastigotes within host macrophages. Their intracellular localization promotes immune evasion and represents a major therapeutic challenge, as effective treatment requires efficient intracellular drug delivery [4]. This feature highlights the need for targeted therapeutic strategies capable of penetrating infected macrophages.

Current antileishmanial therapies, including pentavalent antimonials (SbV), amphotericin B, paromomycin, and oral drug miltefosine, are limited by severe adverse effects, high costs, prolonged treatment regimens, and the emerging drug resistance [5]. These constraints underscore the urgent need for safer and more effective therapeutic alternatives, particularly for CL.

Among natural bioactive sources, snake venom has attracted increasing attention due to its pharmacologically diverse components with antimicrobial and antiprotozoal potential [6]. Snake venoms are complex mixtures of proteins, peptides, and enzymes with potent biological activity. Beyond their defensive function, venom-derived molecules have demonstrated antimicrobial, anticancer, and antiparasitic effects in experimental studies [7]. These bioactive constituents may impair parasite survival through membrane destabilization, oxidative stress induction, and disruption of intracellular signaling pathways, ultimately reducing parasite viability or infectivity.

Venom from the desert black snake *Walterinnesia aegyptia* contains multiple bioactive components that may contribute to antiparasitic activity, including phospholipase A_2_ (PLA_2_), L-amino acid oxidases (LAAOs), disintegrins, and metalloproteinases [8]. These molecules can disrupt parasite membranes, generate reactive oxygen species, and interfere with parasite adhesion and host–cell invasion, suggesting a promising therapeutic potential against intracellular protozoan infections such as cutaneous leishmaniasis.

Given the inherent cytotoxicity of crude venom, optimization of its pharmaceutical properties is essential for safe therapeutic application. Recent developments in engineered nanoplatforms have highlighted the ability of nanocarrier systems to improve biomolecule stability, targeted delivery, and therapeutic efficiency in complex biological environments, thereby supporting their application in advanced biomedical therapies [9]. Nanotechnology-based delivery systems offer a rational strategy to enhance stability, enable controlled release, improve intracellular targeting, and reduce systemic toxicity [10].

In addition, nanoparticle-based delivery platforms can improve drug bioavailability, protect sensitive biomolecules from degradation, and facilitate targeted delivery to diseased tissues [11,12,13,14,15]. These advantages have expanded the biomedical applications of nanomedicine and support the integration of natural bioactive compounds, including venom-derived molecules, into advanced therapeutic platforms designed to overcome current treatment limitations.

In this context, the present study extends recent developments in biomaterial-based nanotechnology by incorporating *Walterinnesia aegyptia* venom into both organic hyaluronic acid-based and inorganic silver nanoparticle-based delivery systems for experimental cutaneous leishmaniasis.

In this study, *Walterinnesia aegyptia* venom was formulated using two nanotechnological strategies: (i) encapsulation within hyaluronic acid-based nanoparticles (HA NPs) and (ii) conjugation with silver nanoparticles (Ag-NPs). HA NPs were selected due to biocompatibility, biodegradability, and structural similarity to endogenous polysaccharides, which can enhance stability, prolong circulation, and improve targeting to inflamed or infected tissues [16,17]. Ag NPs were employed for their intrinsic antimicrobial properties, high surface reactivity, and unique physicochemical characteristics, which may enhance therapeutic efficacy through synergistic antiparasitic activity, improved cellular uptake, and increased stability [18].

To the best of our knowledge, this study represents the first investigation of nanoformulated *Walterinnesia aegyptia* venom for the treatment of cutaneous leishmaniasis, together with a direct comparison between organic (hyaluronic acid-based) and inorganic (silver nanoparticle-based) nanocarrier platforms. This approach not only introduces a novel venom-based nanotherapeutic strategy but also provides important insights into how nanocarrier composition may influence intracellular targeting, therapeutic efficacy, and safety in a parasitic infection model.

Accordingly, the present study evaluates the therapeutic efficacy and safety of *Walterinnesia aegyptia* venom delivered via hyaluronic acid-based nanoparticles and silver nanoparticle conjugates in a murine model of cutaneous leishmaniasis, with the aim of determining the most effective nanocarrier platform for antileishmanial therapy.

## 2. Materials and Methods

### 2.1. Venom Extraction

The crude venom of adult *Walterinnesia aegyptia* was generously gifted by Dr. Mohamed K. Al-Sadoon. All procedures involving live snakes were performed in accordance with the guidelines approved by the Institutional Review Board (IRB), King Saud University, as described previously [19,20]. Briefly, snakes were maintained in a controlled serpentarium under standard husbandry conditions, including temperature regulation using a 100-watt heating lamp with a 9 h/day photoperiod and continuous access to water. Venom was collected from adult snakes via manual milking of the venom glands, pooled, and immediately lyophilized. The lyophilized venom was reconstituted in sterile phosphate-buffered saline (PBS) to a stock concentration of 10 mg/mL, sterilized through a 0.22 μm membrane filter, and stored at −20 °C.

### 2.2. In Vitro Culture of Leishmania Parasites

Isolation and culture of *Leishmania major* were carried out as previously described and in accordance with the guidelines approved by the Committee of Research Ethics, Deanship of Scientific Research, Qassim University, Saudi Arabia (Approval No. 20-03-02/30 September 2020) [21]. Cryopreserved *Leishmania major* promastigotes (Qassim strain, Saudi Arabia) were thawed and cultured in Schneider’s Insect Medium (Invitrogen, Carlsbad, CA, USA) supplemented with 10% (*v*/*v*) heat-inactivated fetal bovine serum (FBS; UFC Biotech, Riyadh, Saudi Arabia) and 100 U/mL penicillin:100 μg/mL streptomycin (Thermo Fisher Scientific Inc., Mississauga, ON, Canada) at 26 °C in 25 cm^2^ tissue culture flasks [22]. Logarithmic-phase promastigotes were harvested by centrifugation at 3000 rpm for 20 min prior to experimental use.

### 2.3. Dose–Response Curve of Walterinnesia aegyptia Venom Against Leishmania Major Using MTT Assay

The antileishmanial activity of *Walterinnesia aegyptia* venom was evaluated against *Leishmania major* promastigotes using the MTT colorimetric assay. Promastigotes 1 × 10^6^ cells/mL were cultured in appropriate growth medium and seeded into 96-well plates at a defined parasite density (200 µL per well). The parasites were then exposed to increasing concentrations of venom (0–200 µg/mL). Amphotericin B (Amphot B) was applied as a positive control and incubated under standard culture conditions.

After incubation, MTT solution was added to each well, and the plates were incubated to allow the formation of formazan crystals. The crystals were dissolved using an appropriate solvent, and absorbance was measured at 570 nm using a microplate reader (xMark, Bio-Rad, Hercules, CA, USA). Parasite viability was calculated relative to untreated controls, and percentage inhibition was determined.

$$\mathrm{Inhibition}\;(\%)=\frac{\left(\mathrm{Viability}\;\mathrm{of}\;\mathrm{control}-\mathrm{Viability}\;\mathrm{of}\;\mathrm{treated}\right)}{\mathrm{Viability}\;\mathrm{of}\;\mathrm{control}}\times 100$$

The IC_50_ value was calculated from the dose–response curve using nonlinear regression analysis or was determined by linear interpolation between the concentrations immediately above and below 50% inhibition, using the formula [23,24].

$$IC_{50}=X_{1}+\frac{\left(50-Y_{1}\right)}{\left(Y_{2}-Y_{1}\right)}\times \left(X_{2}-X_{1}\right)$$

where *X*_1_ and *X*_2_ represent the lower and higher concentrations flanking 50% inhibition, respectively, and *Y*_1_ and *Y*_2_ correspond to the percentage inhibition at *X*_1_ and *X*_2_, respectively.

### 2.4. Flow Cytometric Assessment of Apoptosis via Annexin V/Propidium Iodide Staining

Apoptosis in *Leishmania major* promastigotes was evaluated using the Annexin V-FITC/Propidium Iodide (PI) Apoptosis Detection Kit ThermoFisher Scientific, Invitrogen, catalog number V13242, (Bend, OR, USA) and the flow cytometer analyzer FACSAria III (BD Biosciences Becton, Dickinson and Company, Franklin Lakes, NJ, USA), which detects phosphatidylserine externalization on the cell membrane as an early marker of apoptosis [25].

Logarithmic-phase promastigotes (1 × 10^6^ cells) were cultured in phenol red-free Schneider’s Drosophila medium supplemented with 10% heat-inactivated fetal bovine serum (FBS), 100 IU/mL penicillin, and 100 µg/mL streptomycin. Cultures were maintained at 26 °C under sterile incubation conditions. Parasites were harvested by centrifugation (500× *g*, 5 min, 4 °C), washed twice with phosphate-buffered saline (PBS), and resuspended at a density of 2.5 × 10^5^ cells/mL in 24-well plates (0.5 mL per well).

Promastigotes were treated with crude *Walterinnesia aegyptia* venom at concentrations of 12.5, 25, 50, 100, and 200 µg/mL, with untreated cultures serving as negative controls. After 72 h of incubation, cells were collected and stained with Annexin V-FITC and PI according to the manufacturer’s instructions. Stained cells were analyzed by flow cytometry to quantify viable, early apoptotic, late apoptotic, and necrotic populations, thereby assessing the apoptosis-inducing effect of crude venom on *L. major* promastigotes [26].

### 2.5. Preparation of Hyaluronic Acid-Encapsulated Walterinnesia aegyptia Venom Nanoparticles

Hyaluronic acid (HA)-encapsulated *Walterinnesia aegyptia* venom nanoparticles (WA-HA NPs) were synthesized using a modified chemical crosslinking method based on the procedure described previously [27]. Briefly, 1 g of HA was dissolved in 100 mL of phosphate buffer (pH 7.4) under continuous magnetic stirring until complete dissolution was achieved. Subsequently, 2 mL of the prepared venom stock solution (40 µg/mL) was added to the HA solution and stirred for 1 h to ensure homogeneous mixing. The pH of the reaction mixture was then adjusted to 7.0 using 0.05 M NaOH.

Glutaraldehyde was subsequently added as a crosslinking agent at a final concentration of 0.5% (*v*/*v*) of the reaction mixture, and the mixture was incubated at 37 °C for 72 h under continuous stirring to allow nanoparticle formation.

The resulting WA-HA nanoparticles were purified by dialysis using regenerated cellulose dialysis tubing (molecular weight cut-off [MWCO]: 12–14 kDa). Approximately 10–20 mL of the nanoparticle suspension was transferred into the dialysis tubing and dialyzed against 1 L of deionized water at 4 °C under gentle magnetic stirring (~200 rpm) for 10 h. The external dialysis medium was replaced every 2 h to ensure efficient removal of unreacted components and residual glutaraldehyde. After dialysis, the purified WA-HA nanoparticles were collected and stored at 4 °C until further use, as commonly described for polymeric nanoparticle purification [28].

### 2.6. Preparation of Walterinnesia aegyptia Venom-Conjugated Silver Nanoparticles

Silver nanoparticles were synthesized by dissolving 8.5 mg of silver nitrate (AgNO_3_) in 50 mL of distilled water under continuous stirring for 15 min. Subsequently, 5 mL of *Walterinnesia aegyptia* venom extract (pH 7.0) was added dropwise under constant stirring. The reaction mixture was maintained at room temperature until a visible color change from pale yellow to brown indicated nanoparticle formation. Formation of WA–Ag nanoparticles was further confirmed by physicochemical characterization (Section 2.7), as commonly described for green synthesis of silver nanoparticles [29].

The resulting suspension was centrifuged at 3000 rpm for 20 min and washed three times with distilled water. Purified WA-Ag nanoparticles were resuspended in distilled water and stored at 4 °C until further use. No external chemical reducing agent was used during the synthesis process. Reduction of silver ions was mediated by bioactive components present in the venom, which also contributed to nanoparticle stabilization.

### 2.7. Characterization of WA-HA NPs and WA-Ag NPs

Surface morphology of WA–HA and WA–Ag nanoparticles was assessed using scanning electron microscopy (SEM; JEOL JSM-7600F, 15–20 kV; JEOL, Tokyo, Japan) after mounting samples on aluminum stubs, air-drying, and gold sputter-coating prior to imaging. Transmission electron microscopy (TEM; TEM-1011, 40–100 kV; JEOL, Tokyo, Japan) was used to determine particle size and morphology [30]. Elemental composition was analyzed using energy-dispersive X-ray spectroscopy (EDS; EDAX Octane Silicon Drift Detector, 15–20 kV; EDAX, Ametek, Berwyn, PA, USA), confirming the presence of organic elements (C, O, N) in WA–HA nanoparticles and metallic silver in WA–Ag nanoparticles, thereby verifying successful nanoparticle formation [31]. The hydrodynamic size, zeta potential, and polydispersity index (PdI) of the WA-based nanoparticles were determined using dynamic light scattering (DLS) analysis performed using a Zetasizer (ZEN3600, Malvern Nano Series, Malvern, Worcestershire, UK). All analyses were performed at the Central Research Laboratory, King Saud University.

### 2.8. Experimental Design and In Vivo Treatment

All experimental procedures involving the leishmaniasis murine model were conducted in accordance with the institutional guidelines for the care and use of laboratory animals and were approved by the IRB, King Saud University (Ethical Approval No. KSU-SE-22-103). All efforts were made to minimize animal suffering and to reduce the number of animals used throughout the study while obtaining a sufficient number for significant statistical tests.

*Leishmania major* promastigotes were cultured in Schneider’s Insect Medium until reaching the stationary growth phase. The parasite pellet was collected by centrifugation and resuspended in sterile buffer to obtain the desired concentration for murine infection.

Sixty 8-week-old female BALB/c mice were obtained from the Female Centre for Scientific and Medical Colleges, King Saud University, Riyadh, Saudi Arabia. Each mouse was subcutaneously inoculated at the dorsal tail base with 0.1 mL of *Leishmania major* promastigote suspension containing 1 × 10^7^ parasites/mL, as previously described [32].

After the appearance of lesions, mice were randomly divided into six experimental groups (n = 10 per group) as follows: Group I: non-infected healthy control mice, Group II: infected untreated control mice, Group III: infected mice treated intraperitoneally with Amphot B (1 mg/kg/day), Group IV: infected mice treated orally with 0.2 mg/kg/day crude *Walterinnesia aegyptia* venom (WA), Group V: infected mice treated orally with WA encapsulated in hyaluronic acid nanoparticles (WA-HA NPs), and Group VI: infected mice treated orally with WA silver nanoparticles (WA-Ag NPs).

Treatment was initiated upon the first appearance of ulcerative lesions and continued for seven consecutive days. Mortality was monitored daily throughout the experimental period. Lesion progression was monitored for four weeks after treatment to evaluate therapeutic efficacy [33,34].

Lesion size was measured weekly using a Vernier caliper by recording two perpendicular diameters (a and b). The lesion size (LS) was calculated according to the following formula:

$$LS=\frac{a\times b}{2}$$

At the end of the experiment, mice were sacrificed by decapitation in accordance with the institutional Animal Ethics Committee guidelines. Skin tissues from lesion sites, as well as liver and spleen samples, were collected. Samples intended for molecular analysis were snap-frozen in liquid nitrogen and stored at −80 °C until further processing.

### 2.9. Measuring Liver Enzymes

Serum levels of alanine aminotransferase (ALT), aspartate aminotransferase (AST), and alkaline phosphatase (ALP) were measured using commercial spectrophotometric kits (Spectrum Diagnostics, Cairo, Egypt), according to the manufacturer’s instructions.

### 2.10. Histological Examination

Liver and spleen tissues were immediately collected and fixed in 10% neutral-buffered formalin for 24 h, followed by routine tissue processing and paraffin embedding. Sections were prepared at 5 µm thickness and stained with hematoxylin and eosin (H&E) for histopathological examination according to standard protocols [35]. Photomicrographs were obtained using a Nikon light microscope (Eclipse E200-LED, Tokyo, Japan).

### 2.11. Molecular Detection of Leishmania Major in Mouse Lesions

Genomic DNA was extracted from skin lesion tissues using the DNeasy Blood & Tissue Kit (Qiagen, Hilden, Germany) according to the manufacturer’s instructions. DNA concentration and purity were determined spectrophotometrically, and samples were stored at −20 °C until analysis.

Detection of *Leishmania major* DNA was performed by PCR amplification of the kinetoplast DNA (kDNA) minicircle region (600 bp) using primers RV1 (5′-CTTTTCTGGTCCCGCGGGTAGG-3′) and RV2 (5′-CCACCTGGCCTATTTTACACCA-3′) as previously described [36]. PCR products were separated by 1.5–2% agarose gel electrophoresis, stained with ethidium bromide, and visualized under UV illumination. A 100 bp DNA ladder was used as a molecular size marker. Samples showing a distinct band at 600 bp were considered positive for *L. major* DNA [37,38].

### 2.12. Statistical Analysis

Data are presented as mean ± standard deviation (SD). Statistical analyses were performed using SPSS software (Version 22.0; IBM Corp., Armonk, NY, USA). The normality of data distribution was assessed prior to analysis. Comparisons between two groups were conducted using Student’s *t*-test, while comparisons among multiple groups were conducted using ANOVA followed by Tukey’s post hoc test for multiple comparisons. A *p*-value ≤ 0.05 was considered statistically significant.

## 3. Results

### 3.1. Anti-Promastigote Activity and Apoptosis Induction by Walterinnesia aegyptia Venom

**Figure 1:**
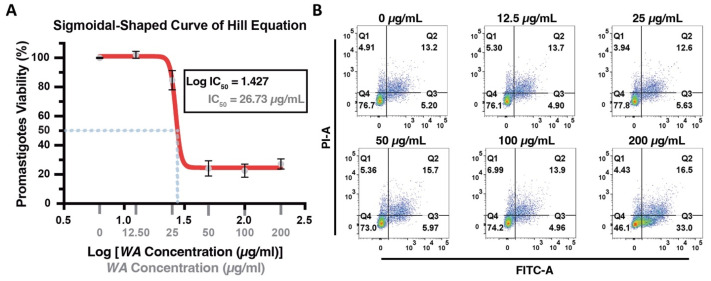
Anti-promastigote activity and apoptosis-inducing effect of WA venom. (**A**) Semi-logarithmic dose–response curve showing the effect of crude *Walterinnesia aegyptia* venom (*WA*) on promastigote viability. Data were fitted using a four-parameter logistic (Hill) equation, yielding a sigmoidal (S-shaped) curve and an IC_50_ of 26.73 µg/mL. (**B**) Flow cytometric analysis of apoptosis in *Leishmania major* promastigotes following 72 h exposure to *WA*. Annexin V-FITC/propidium iodide (PI) staining distinguishes viable, early apoptotic, late apoptotic, and necrotic populations in gated low-forward scatter (FSC) promastigote cells enriched for metacyclic forms. Each dot plot is divided into four quadrants representing distinct cell-death phenotypes based on fluorescence: Q1, Annexin V^−^/PI^+^ (necrotic cells); Q2, Annexin V^+^/PI^+^ (late apoptotic cells); Q3, Annexin V^+^/PI^−^ (early apoptotic cells); Q4, Annexin V^−^/PI^−^ (viable cells).

To evaluate the antileishmanial potential of crude *Walterinnesia aegyptia* (*WA*) venom, *Leishmania major* promastigotes were exposed to increasing venom concentrations, and parasite viability was assessed using the MTT assay (Figure 1A). WA venom induced a clear concentration-dependent reduction in promastigote viability, indicating pronounced cytotoxic activity. A sharp decline in viability was observed at lower concentrations, reflecting the high sensitivity of promastigotes to WA venom. At higher concentrations (>50 µg/mL), the inhibitory effect reached a plateau, with only minimal fluctuations in viability.

Nonlinear regression analysis of the dose–response curve yielded an IC_50_ value of 26.73 µg/mL, indicating that this concentration reduces parasite metabolic activity by 50%. Interestingly, substantial inhibition was already evident at relatively low concentrations by about ~20 µg/mL, where viability approached the IC_50_ threshold. Together, these findings demonstrate that WA venom exerts potent, dose-dependent cytotoxic effects on promastigotes, supporting its potential as a promising antileishmanial agent through the disruption of parasite metabolic activity.

To investigate the mechanism underlying parasite death, Annexin V-FITC/propidium iodide (PI) flow cytometry was performed to distinguish viable, apoptotic, and necrotic cells following WA treatment (Figure 1B). Untreated promastigotes exhibited high baseline viability (76.7%), with the majority of cells located in the Q4 quadrant (Annexin V^−^/PI^−^), indicating intact membrane integrity and minimal apoptotic activity. With increasing concentrations of WA venom, a progressive shift in the cell population from the viable quadrant toward the apoptotic quadrants was observed.

At lower concentrations (12.5–100 µg/mL), only modest changes in cell survival were detected, and no statistically significant reduction in viability was observed (*p* ≈ 0.1), suggesting limited cytotoxicity at sub-maximal doses. However, treatment with 200 µg/mL WA venom resulted in a marked decrease in viability to 46.1% (*p* = 0.0004). This reduction was accompanied by a significant increase in early apoptotic cells, representing an approximately 5 ± 1.9-fold elevation compared with untreated controls (*p* = 0.011). Late apoptotic populations showed a gradual but non-significant increase, whereas necrotic cells remained relatively low across all concentrations.

When early and late apoptotic populations were combined, total apoptosis increased approximately 3.98 ± 3.1-fold relative to untreated controls, while overall cell death increased 2.28 ± 0.9-fold (*p* = 0.0398). These findings indicate that WA venom induces apoptosis-mediated cytotoxicity in *L. major* promastigotes, with apoptosis representing the predominant mechanism of parasite death rather than nonspecific necrosis.

Overall, the dose–response cytotoxicity assay and flow-cytometric analysis collectively demonstrate that WA venom possesses potent antileishmanial activity and triggers apoptosis-like programmed cell death in *Leishmania major* promastigotes in a concentration-dependent manner (Figure 1).

### 3.2. Characterization of WA-Based Nanoparticles

**Figure 2:**
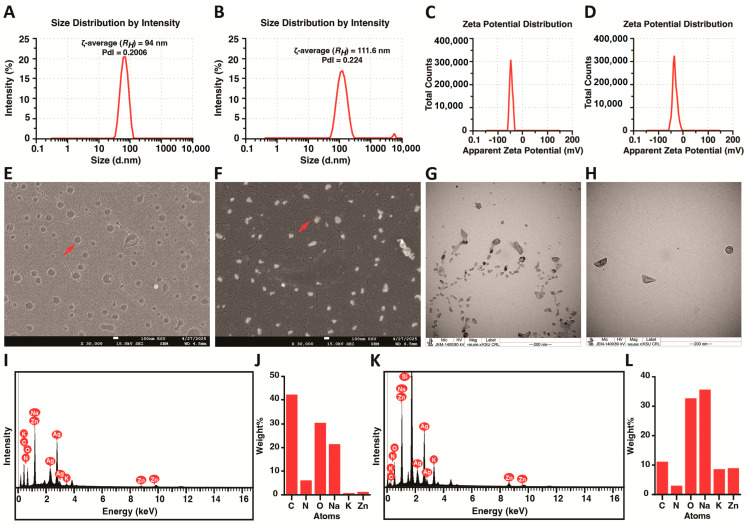
Physicochemical characterization of WA-based nanocarrier systems. (**A**,**B**) DLS analyses of WA-HA and WA-Ag, respectively, to determine the hydrodynamic diameter presented by zeta-average (nm) on a logarithmic scale on the X-axis versus the intensity (%) on the Y-axis. (**C**,**D**) Zeta potential distribution of WA-HA and WA-Ag, respectively, demonstrating the average surface charges in mVolt (apparent Zeta Potential, mV). (**E**) SEM image of WA-HA showing smooth, spherical nanostructures typical of hyaluronic acid-based carriers. (**F**) SEM image of WA-Ag showing dense metallic nanostructures with well-defined morphology. Scale bar = 100 nm. (**G**) TEM image of WA-HA demonstrating small nanoparticle size and irregular nanostructures. (**H**) TEM image of WA-Ag showing larger and more uniform nanoparticles. Scale bar = 200 nm. (**I**) EDS spectrum of WA-HA showing the elemental composition (C, O, N, Na, K, Zn) together with low- to medium-intensity Ag signals. keV = kilo-electronvolt. (**J**) Elemental distribution profile of WA-HA NPs. (**K**) EDS spectrum of WA-Ag showing higher characteristic Ag peaks, consistent with the presence of silver nanoparticles. (**L**) Elemental distribution profile of WA-Ag NPs.

DLS and zeta potential analyses were conducted to evaluate the physical characteristics and colloidal stability of the synthesized nanoparticles. The hydrodynamic diameter of the WA-HA NPs was measured at 94 nm with a polydispersity index (PdI) of 0.2006 (Figure 2A). In comparison, the WA-Ag NPs exhibited a larger hydrodynamic size of 111.6 nm and a PdI of 0.224 (Figure 2B). The Zeta potential values for WA-HA NPs and WA-Ag NPs were recorded at −40 mV and −39.2 mV, respectively (Figure 2C,D). These high negative magnitudes indicate strong electrostatic repulsion between the particles, suggesting excellent long-term colloidal stability. Such physical robustness is critical for ensuring the efficacy of these nano-formulations in biomedical applications, particularly as targeted therapeutic agents for cutaneous leishmaniasis.

Additionally, scanning electron microscopy (SEM), transmission electron microscopy (TEM), and energy-dispersive X-ray spectroscopy (EDS) were used to characterize the morphology and elemental composition of the synthesized WA-based nanoparticles. SEM analysis (Figure 2E,F) revealed distinct morphological differences between the two nanoparticle formulations. The WA-HA nanoparticles (Figure 2E) appeared as smooth, rounded particles distributed uniformly across the surface, indicating successful encapsulation of *Walterinnesia aegyptia* venom within the hyaluronic acid matrix. In contrast, the WA-Ag nanoparticles (WA-Ag NPs) (Figure 2F) showed bright electron-dense particles, characteristic of metallic silver nanoparticles conjugated with the venom. These SEM images were obtained at 30,000× magnification, confirming the nanoscale structure of both formulations.

TEM imaging provided further insight into particle size and structural morphology (Figure 2G,H). The WA-HA nanoparticles (Figure 2G) appeared as smaller, irregularly shaped particles, with sizes ranging approximately from 20 to 56 nm. In comparison, the WA-Ag NPs (Figure 2H) were larger and more defined, with particle diameters ranging approximately from 70 to 120 nm, confirming the successful formation of silver-based nanoparticles.

Elemental composition analysis was performed using energy-dispersive X-ray spectroscopy (EDS) (Figure 2I–L). The EDS spectrum of WA-HA NPs (Figure 2I) showed dominant peaks corresponding to carbon (C), oxygen (O), and nitrogen (N), which are characteristic elements of organic polysaccharide-based nanostructures such as hyaluronic acid and venom proteins. The corresponding elemental quantification chart (Figure 2J) confirmed that carbon and oxygen were the major constituents, while nitrogen appeared at lower levels. The detection of sodium (Na) likely reflects the sodium-hyaluronate form of HA, whereas trace amounts of potassium (K) and zinc (Zn) may originate from residual salts.

In contrast, the EDS spectrum of WA-Ag NPs (Figure 2K) exhibited distinct peaks corresponding to metallic silver (Ag), confirming the successful incorporation of silver within the venom-based nanoparticle system. Additional peaks corresponding to carbon, oxygen, nitrogen, and sodium were also observed, reflecting the organic components of the venom matrix. The elemental composition chart (Figure 2L) further demonstrated the presence of these elements, while the detection of silver confirmed the successful synthesis of WA-Ag NPs.

Overall, the SEM, TEM, and EDS analyses collectively confirm the successful fabrication of WA-HA and WA-Ag nanoparticle systems, with distinct morphological and elemental characteristics.

### 3.3. Therapeutic Efficacy of WA Venom and Nanoparticle Formulations in L. major-Infected Mice

**Figure 3:**
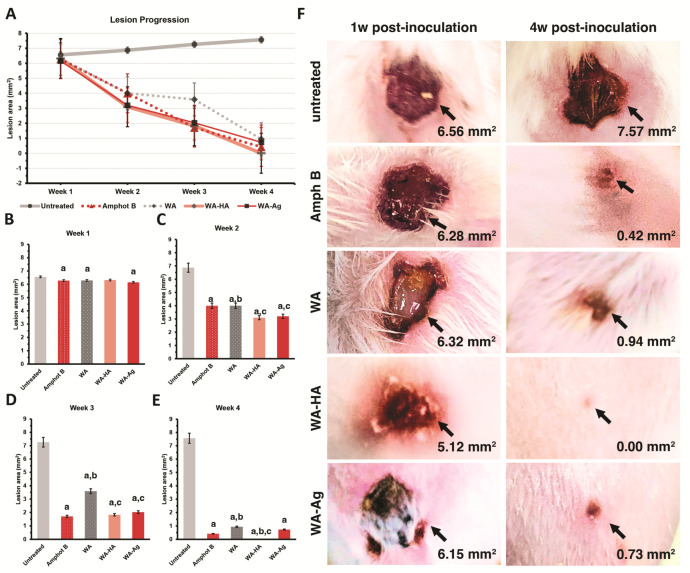
In vivo therapeutic efficacy of *Walterinnesia aegyptia* venom in experimental cutaneous leishmaniasis. (**A**) Time-dependent changes in lesion size across all animal groups over the 4-week experimental period, showing a distinct therapeutic response among treatments. (**B**) Lesion size 1-week post-treatment, showing comparable lesion dimensions among infected groups. (**C**) Lesion size 2 weeks post-treatment, demonstrating clear reduction in all treated groups compared with untreated animals. (**D**) Lesion size 3 weeks post-treatment, showing a stronger therapeutic response in WA-HA and WA-Ag compared with WA alone. (**E**) Lesion size 4 weeks post-treatment, demonstrating complete to near-complete lesion regression in WA-, WA-HA and WA-Ag groups, respectively. Data in (**B**–**E**) are presented as mean ± SD. Statistical significance when *p*-values are ≤0.05 in a comparison with parasite-free a, with untreated b, and with Amphot B-treated c. (**F**) Representative macroscopic images showing progressive lesion enlargement in untreated animals and marked lesion healing in WA, WA-HA-, and WA-Ag-treated groups.

The therapeutic efficiency of *Walterinnesia aegyptia* (*WA*) venom and nanoparticle formulations was assessed in a *Leishmania major*-infected BALB/c mouse model by measuring the lesion progression over four weeks after treatment (Figure 3). Lesion size was measured weekly using a Vernier caliper, and both quantitative measurements and macroscopic observations were recorded.

In the untreated infected control group, lesions developed progressively after infection and continued to enlarge throughout the experimental period (Figure 3A). Initially, lesions appeared as localized swelling and erythema that subsequently progressed to ulcer formation and tissue deterioration. By week 4, untreated mice exhibited severe disease progression characterized by pronounced ulceration and markedly thickened lesion rim margins around 4 weeks post-treatment (Figure 3B), with an average lesion magnification of about 7.6 mm^2^ at that point (Figure 3F).

In contrast, progressive lesion regression was seen over time for all treatment groups. Lesion sizes in treated mice were significantly smaller than in untreated animals from week 2 onwards (*p* < 0.05), as shown graphically (Figure 3B–E) and quantitatively. This improvement was also visible macroscopically, as treated lesions showed less erythema in the peripheral area and decreased swelling followed by progressive drying of ulcerative parts.

Mice treated with crude WA venom demonstrated gradual lesion regression during the treatment period. By week 4, lesion size decreased to approximately 0.94 mm^2^, indicating partial healing with a small residual lesion. More pronounced therapeutic effects were observed in mice treated with WA-HA NPs, WA-Ag NPs, and Amphotericin B. These groups exhibited accelerated lesion contraction during weeks 3 and 4, indicating stronger therapeutic responses.

Notably, the WA-HA NP-treated group showed the most effective lesion regression, with complete lesion resolution by week 4 (0.00 mm^2^) and restoration of nearly normal skin appearance. Similarly, Amphotericin B-treated mice showed substantial lesion reduction, reaching approximately 0.42 mm^2^ by the end of the experiment. The WA-Ag NPs group also demonstrated significant lesion healing, with lesions reduced to approximately 0.73 mm^2^.

Overall, both quantitative lesion measurements (Figure 3A–E) and macroscopic observations of lesion healing (Figure 3F) confirm that WA venom possesses clear in vivo antileishmanial activity and that nanoparticle-based formulations—particularly WA-HA NPs—significantly enhance therapeutic efficacy, producing treatment outcomes comparable to the standard antileishmanial drug Amphotericin B.

### 3.4. Hepatic Biochemical and Histopathological Recovery Following Treatment

**Figure 4:**
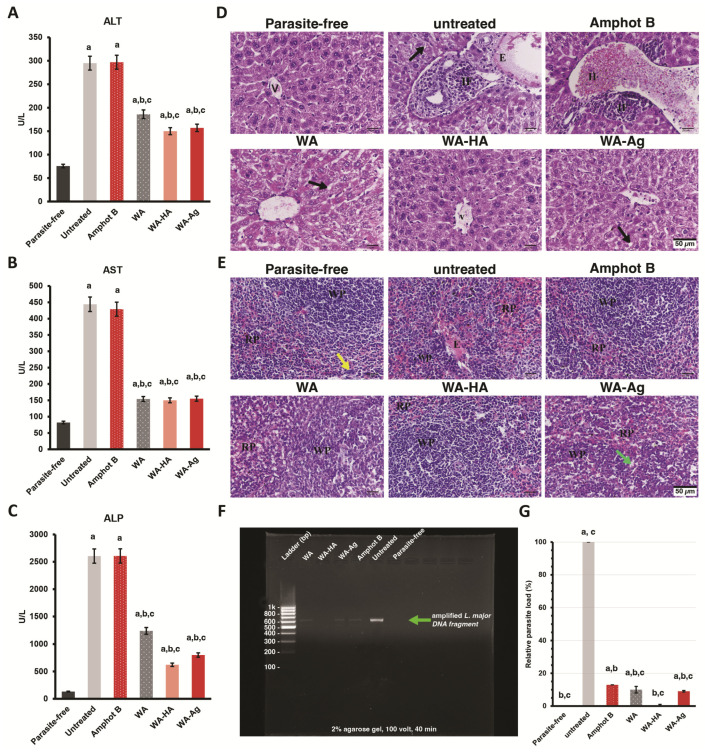
Biochemical recovery, tissue restoration, and molecular confirmation of parasite reduction. Biochemical analysis of serum levels of (**A**) alanine aminotransferase (ALT), (**B**) aspartate aminotransferase (AST), and (**C**) alkaline phosphatase (ALP). *Leishmania major* infection markedly increased liver enzyme levels in untreated animals, whereas treatment with Amphot B, WA, WA-HA, and WA-Ag restored enzyme levels toward normal values. (**D**,**E**) Histopathological evaluation of liver and spleen tissues after 4 weeks of treatment. Scale bar = 50 µm. (**D**) Representative liver sections stained with hematoxylin and eosin (H&E) showing normal hepatic architecture in parasite-free animals with a central vein (V). Untreated infected animals showed marked inflammatory infiltration (IF), edema (E), and cytoplasmic degeneration of hepatocytes (black arrow). Liver sections from infected animals treated with Amphot B showed inflammatory infiltration (IF) and a congested hemorrhagic vein (H). WA-treated animals demonstrated mild steatotic changes (black arrow), whereas WA- Ag NPs treated animals showed partial histological improvement with mild residual steatosis. The greatest restoration of normal hepatic architecture was observed in the WA-HA NPs -treated group, with preservation of hepatocyte organization and a clearly visible central vein (V). V = central vein; E = congestion with edema; H = congestion with hemorrhage; IF = inflammatory infiltration. (**E**) Representative spleen sections (H&E staining) showing normal splenic architecture in parasite-free animals, severe tissue disruption in untreated animals, and varying degrees of splenic tissue recovery in Amphot B, WA, WA-Ag NsP-treated, and WA-HA NPs -treated groups, with the greatest restoration observed in the WA-HA NPs -treated group. WP = white pulp; RP = red pulp; E = edema. The yellow arrow indicates normal splenic macrophages in the parasite-free control group, whereas the green arrow indicates focal necrosis in the WA-Ag -treated group. (**F**,**G**) Molecular confirmation of parasite burden after 4 weeks of treatment. (**F**) Agarose gel electrophoresis of PCR amplification targeting the ~600 bp kinetoplast DNA fragment of *Leishmania major* isolated from murine lesion tissues, showing strong amplification in untreated animals and reduced amplification in WA, WA-HA NP, and WA-Ag NP-treated groups. (**G**) Relative parasite burden (%) measured from gel quantification, showing a significant reduction, approaching absence in WA-HA NP following treatment. Data in (**A**,**B**,**C**,**G**) are presented as mean ± SD. Statistical significance when *p*-values are ≤0.05 for a comparison to parasite-free, b to untreated, and c to Amphot B-treated.

To determine whether treatment with WA venom and its nanoparticle formulations could reduce infection-associated tissue damage, hepatic enzyme activities together with histopathological alterations in liver and spleen tissues were evaluated at the experiment endpoint (Figure 4).

*Leishmania major* infection caused marked hepatic dysfunction, as demonstrated by significant elevation in ALT, AST, and ALP levels in the untreated group compared with the uninfected control (*p* < 0.05; Figure 4A–C). These findings confirm that systemic infection was associated with substantial liver injury. Treatment with Amphot B resulted in only partial normalization of the enzyme levels, which remained significantly higher than those of the uninfected control group. In contrast, treatment with WA venom and its nanoparticle formulations produced a more pronounced reduction in all three liver enzymes, with ALT, AST, and ALP levels approaching normal ranges. Among the tested formulations, WA-HA NPs showed the strongest improvement in hepatic biochemical parameters (Figure 4A–C).

Histopathological examination supported these biochemical findings. Liver sections from the control group displayed normal architecture characterized by well-organized hepatocytes and intact vascular structures (Figure 4D). In contrast, untreated infected mice showed marked pathological alterations, including inflammatory cell infiltration, edema, and hepatocellular degeneration. Treatment with WA venom, WA-HA NPs, and WA-Ag NPs resulted in clear histological recovery, with well-preserved hepatocytes and a marked reduction in inflammatory infiltration.

To further verify parasite clearance, PCR amplification of the kinetoplast DNA (kDNA) minicircle region of *L. major* was performed on lesion tissue samples (Figure 4F). Untreated infected mice showed a strong amplification band at approximately 600 bp, confirming the presence of parasite DNA and active infection. In contrast, treated groups exhibited markedly reduced band intensity, indicating a considerable decrease in parasite load after treatment. However, WA-HA and WA-Ag nanoparticle formulations showed the weakest amplification signals, while the Amphotericin B treatment group exhibited moderate band intensity.

Quantitative analysis of PCR band intensity confirmed these observations (Figure 4G). Untreated infected mice exhibited the highest relative parasite load, while all treatment groups showed significant reductions in parasite DNA levels. The lowest parasite burden was detected in the WA-HA NPs group, followed by WA-Ag NPs and crude WA venom.

Collectively, these biochemical, histopathological, and molecular findings demonstrate that WA venom treatment effectively reduces parasite burden and mitigates infection-induced tissue damage, with nanoparticle formulations particularly providing the most pronounced therapeutic benefit.

## 4. Discussion

Leishmaniasis remains a major global health problem due to the limited efficacy, toxicity, and increasing resistance associated with conventional antileishmanial drugs. In addition, many currently available treatments exhibit poor intracellular efficacy, leading to relapse and persistent infection [39]. Consequently, there is growing interest in developing alternative therapeutic strategies, particularly those based on natural bioactive compounds and nanotechnology-based drug delivery systems [40,41]. Natural products derived from diverse biological sources represent a valuable reservoir for the discovery of novel therapeutics to combat drug-resistant infections. Sources such as plants, algae, and animal venoms have gained considerable attention due to their rich content of bioactive compounds with antimicrobial, cytotoxic, and anti-inflammatory properties [42,43]. Compared with synthetic drugs, natural products are often more accessible and may exhibit fewer adverse effects.

Among these, animal venoms are particularly noteworthy due to their biochemical diversity and evolutionary role as defense and predation tools. Importantly, venom-derived molecules have demonstrated broad-spectrum antimicrobial activity against bacteria, fungi, viruses, and parasites, highlighting their potential as promising candidates for therapeutic development [44,45]. However, snake venoms have attracted considerable attention due to their rich composition of biologically active molecules with therapeutic potential. Research on venom components is essential for developing targeted therapeutic strategies due to their specificity and potency [46].

Recent studies have demonstrated that snake venoms represent a promising source of antiparasitic agents, containing a wide range of bioactive molecules such as phospholipases A_2_, metalloproteinases, L-amino acid oxidases, and antimicrobial peptides, which can disrupt parasite membranes and interfere with essential cellular processes [47,48]. These molecules exert their effects through mechanisms including membrane disruption, oxidative stress induction, enzymatic degradation, and immune modulation, ultimately leading to parasite death [49]. Several studies have confirmed the antileishmanial activity of venom-derived compounds, demonstrating their ability to inhibit parasite proliferation, induce apoptosis-like cell death, and reduce infectivity in *Leishmania* species [50].

The pro-apoptotic effects observed in the present study may be attributed, at least in part, to enzymatic components of *Walterinnesia aegyptia* venom, particularly phospholipase A_2_ (PLA_2_) and L-amino acid oxidases (LAAOs). These enzymes are well-recognized for their cytotoxic and pro-apoptotic activities, including membrane disruption, mitochondrial dysfunction, and induction of oxidative stress [51]. LAAOs, in particular, generate hydrogen peroxide as a byproduct, which can trigger apoptosis through oxidative damage pathways. The flow cytometry results obtained in this study, showing increased early and late apoptotic populations following venom treatment, are consistent with these mechanisms. Moreover, nanoformulation may further enhance these effects by protecting bioactive proteins from degradation, improving intracellular delivery, and sustaining exposure of parasite cells to active venom components.

In agreement with these reports, the present study demonstrates that *Walterinnesia aegyptia* venom exhibits significant antileishmanial activity against *Leishmania major*, both in vitro and in vivo. The cytotoxic effects observed in the MTT assay are consistent with previous findings indicating that venom-derived peptides and enzymes possess potent antiparasitic activity acting through mechanisms such as membrane disruption, oxidative stress induction, and enzymatic degradation of parasite structures, thereby supporting their potential as novel therapeutic agents for the treatment of leishmaniasis [52].

In addition, the findings of apoptosis-like cell death in this study reinforce previous data supporting the antileishmanial effect of *Walterinnesia aegyptia* venom. Flow cytometric analysis confirmed that the cytotoxic effect was primarily executed through triggering apoptosis, which was evidenced by a significant increase in both early and late apoptotic populations at higher concentrations [53]. Protozoan parasites such as Leishmania are known to undergo apoptosis-like processes characterized by phosphatidylserine externalization, mitochondrial dysfunction, and DNA fragmentation [54]. These events are typically triggered by bioactive molecules, including antimicrobial peptides and toxins, which disrupt mitochondrial function or induce oxidative stress. Similar apoptosis-mediated mechanisms have been reported for other venom-derived compounds with antiparasitic activity [55]. Notably, apoptosis induction, rather than necrosis, would create a better therapeutic strategy by allowing controlled parasite removal and decreased host inflammation.

Another key finding of the current study is the improved treatment efficacy offered by nanoparticle-mediated designs of venom formulations. In regard to drug delivery in parasitic diseases, nanotechnology has been identified as a market entry strategy for enhancing the stability and bioavailability of therapeutic agents while providing targeted delivery [56]. In leishmaniasis, targeted delivery is particularly critical because *Leishmania* parasites reside primarily within macrophages, which serve as their intracellular niche. Nanoparticles have previously been utilized to increase intracellular drug accumulation in infected macrophages, which resulted in improved parasite clearance and increased therapeutic efficacy [57].

Moreover, silver nanoparticles are known for their inherent antimicrobial activity, and hyaluronic acid-based systems may enhance nanoparticle stability, biocompatibility, and targeted delivery [58]. These properties may all add together to explain the superior therapeutic performance of the nanoformulated venom systems that are observed.

Particle size also plays a critical role in determining nanoparticle biodistribution, cellular uptake, and clearance [59]. It has been reported that very small nanoparticles (10–20 nm) may evade efficient macrophage uptake, whereas larger particles (>1 µm) are rapidly cleared by the reticuloendothelial system due to aggregation and opsonization [60]. Therefore, nanoparticles within the size range of 20–200 nm are generally considered optimal for efficient intracellular drug delivery, particularly when targeting macrophage-associated pathogens [61,62].

DLS and zeta potential analyses were performed to evaluate the hydrodynamic size distribution and colloidal stability of the synthesized nanoparticles. WA-HA nanoparticles exhibited a mean hydrodynamic diameter of 94 nm with a polydispersity index (PdI) of 0.2006, indicating a relatively uniform particle distribution. In comparison, WA-Ag nanoparticles showed a slightly larger hydrodynamic diameter of 116.6 nm with a PDI of 0.224.

The zeta potential values of WA-HA and WA-Ag nanoparticles were −40 mV and −39.2 mV, respectively. These high negative surface charges indicate strong electrostatic repulsion between particles and suggest good colloidal stability. Such physicochemical characteristics are considered favorable for biomedical nanocarrier systems and may contribute to improved stability, cellular interaction, and therapeutic performance in cutaneous leishmaniasis treatment, consistent with previous reports on nanoparticle-based antileishmanial formulations [63].

In the present study, TEM analysis revealed particle sizes ranging from 20 to 56 nm for WA-HA nanoparticles and 70–120 nm for WA-Ag nanoparticles, indicating that both formulations fall within the optimal nanoscale range for enhanced cellular uptake and intracellular delivery. These size distributions are well suited for endocytic uptake by macrophages, the primary host cells of *Leishmania* parasites, and are consistent with previous studies demonstrating that nanoparticle size and physicochemical properties significantly influence cellular internalization and targeting efficiency in macrophage-based infection models [64,65].

The superior therapeutic performance observed for WA-HA nanoparticles may be attributed, at least in part, to the targeting properties of hyaluronic acid [66,67]. Hyaluronic acid is known to interact with CD44 receptors expressed on macrophages, facilitating receptor-mediated endocytosis and enhancing intracellular accumulation of therapeutic agents within infected cells [68]. This mechanism may contribute to improved delivery of venom-derived bioactive compounds and enhanced parasite clearance.

Moreover, EDS analysis confirmed the successful incorporation of venom components in both formulations, as well as the presence of metallic silver in WA-Ag nanoparticles [69]. The inclusion of silver nanoparticles may provide additional therapeutic benefits through mechanisms such as reactive oxygen species (ROS) generation, membrane disruption, and mitochondrial dysfunction, which have been widely associated with their antimicrobial and antiparasitic activity [70]. These effects may act synergistically with venom-derived bioactive molecules, thereby enhancing overall therapeutic efficacy.

BALB/c mice are highly susceptible to *L. major* infection and develop progressive cutaneous lesions resembling human cutaneous leishmaniasis. Effective antileishmanial therapies are typically associated with rapid lesion contraction and improved tissue healing [71]. A key finding of this study is that, in vivo, nanoparticle-based WA formulations produced significantly enhanced lesion regression compared with crude venom. Notably, WA-HA nanoparticles achieved near-complete lesion resolution by week four, with therapeutic outcomes comparable to those observed in the Amphotericin B-treated group. This improved efficacy may be attributed to enhanced intracellular delivery and sustained release of venom components within parasite-infected macrophages, thereby facilitating more effective parasite clearance and tissue recovery. In addition, hyaluronic acid plays a critical role in dermal tissue repair and maintenance due to its strong water-retention capacity, ability to enhance tissue regeneration, and regulatory effects on inflammation and wound healing [72]. These properties may further contribute to the pronounced lesion healing observed in the WA-HA NPs -treated group.

The biochemical and histopathological findings of the present study further support the protective effects of WA venom, particularly in its nanoparticle formulations. Elevated hepatic enzyme levels, including ALT, AST, and ALP, are commonly associated with systemic *Leishmania* infection as a consequence of inflammatory responses and parasite dissemination to internal organs [73]. In the present study, treatment with WA-HA and WA-Ag nanoparticles restored these enzyme levels to the WA baseline, indicating significant hepatoprotective effects beyond parasite clearance. This biochemical improvement was corroborated by histopathological observations, where untreated animals exhibited marked hepatocellular degeneration, inflammatory cell infiltration, and disruption of splenic architecture. In contrast, nanoparticle-treated groups demonstrated preservation of hepatic structure and substantial restoration of normal spleen architecture, with minimal residual pathology. The recovery of splenic organization, a key component of the reticuloendothelial system involved in parasite clearance, further supports effective macrophage-targeted delivery of the nanoparticle formulations. These findings are consistent with previous studies reporting that effective antileishmanial therapies are associated with normalization of liver enzyme levels and restoration of tissue architecture following reduction in parasite burden [74].

Finally, molecular detection using PCR targeting the kDNA minicircle region confirmed a marked reduction in parasite burden in treated animals. PCR amplification of kDNA is widely recognized as one of the most sensitive and specific methods for detecting a *Leishmania* infection and evaluating treatment efficacy [75]. In the present study, treated groups showed reduced amplification signals compared with untreated controls, with the weakest bands observed in the WA-HA and WA-Ag nanoparticle groups, indicating superior parasite clearance. These findings are consistent with previous reports demonstrating that effective antileishmanial therapies are associated with a significant reduction in detectable parasite DNA following treatment [76].

Collectively, the molecular results corroborate the clinical and histopathological findings, providing strong evidence that WA nanoparticle formulations—particularly WA-HA nanoparticles—enhance antiparasitic efficacy through improved delivery and intracellular targeting. The findings of the current study show that venom from *Walterinnesia aegyptia* possesses considerable antileishmanial activity and that nanoparticle-based delivery systems markedly enhance its therapeutic efficacy. The combined effects of venom bioactivity, apoptosis induction, targeted nanoparticle delivery, and improved tissue recovery highlight the potential of venom-derived nanomedicines as a promising strategy for the treatment of leishmaniasis.

This study, to the best of our knowledge, is the first demonstrating an in vivo investigation showing that nanoformulation of *Walterinnesia aegyptia* venom has enhanced therapeutic efficacy against cutaneous leishmaniasis. Although most previous research addressed only the in vitro activity of venom-derived compounds, this study further refines these findings by confirming the efficiency of those molecules against an experimental murine model.

Further studies are required to isolate and characterize the active venom com-ponents and to evaluate their toxicity, pharmacokinetics, and long-term safety profiles. In addition, refinement of nanoparticle formulations and evaluation in advanced preclinical and clinical models will be necessary to facilitate future clinical translation.

## 5. Conclusions

Nanoformulation of *Walterinnesia aegyptia* venom (WA-HA NPs and WA-Ag NPs) delivers superior anti-leishmanial efficacy compared with crude venom and matches or exceeds amphotericin B in lesion resolution, parasite clearance, and tissue recovery. Nanocarrier-mediated delivery enhances intracellular targeting, maximizes venom bioactivity, and reduces systemic toxicity. These findings position venom-based nanotherapeutics as a novel, potent strategy for cutaneous leishmaniasis, meriting further mechanistic and translational studies, such as elucidating the underlying mechanisms, identifying active venom components, and evaluating safety and efficacy in advanced preclinical and clinical settings.

## Data Availability

The datasets generated during the current study are available from the corresponding author on reasonable request.
